# [(Triethyl­ene­tetra­mine)copper(II)]-μ-cyanido-κ^2^ *N*:*C*-[bis(cyanido-κ*C*)copper(I)]

**DOI:** 10.1107/S1600536812047745

**Published:** 2012-11-24

**Authors:** Peter W. R. Corfield, Scott A. Grillo, Nancy S. Umstott

**Affiliations:** a Department of Chemistry, Fordham University, 441 East Fordham Road, Bronx, NY 10458, USA; b The King’s College, Briarcliff Manor, NY 10510, USA

## Abstract

The title compound, [Cu_2_(CN)_3_(C_6_H_18_N_4_)] or [Cu(trien)(CN)Cu(CN)_2_], where trien is triethyl­ene­tetra­mine, is a mixed-valence complex crystallizing as discrete mol­ecules, with Cu^I^ and Cu^II^ ions linked by a bridging cyanide group. The Cu^II^ ion is in a square-pyramidal coordination environment, with the N atoms of the tetra­dentate trien ligand occupying the basal positions and Cu—N bond lengths in the range 2.028 (4)–2.047 (4) Å. An *N*-bonded cyanide group is in the apical position, with a slightly longer Cu—N bond length of 2.127 (4) Å. The Cu^I^ ion exhibits a trigonal–planar coordination geometry, bonded to the C atoms of the bridging cyanide group and two terminal cyanide groups with Cu—C bond lengths in the range 1.925 (4)–1.948 (5) Å. In the crystal, hydrogen bonding involving the tertiary N—H groups of the trien ligand and N atoms of symmetry-related terminal cyanide groups links mol­ecules into a ribbon extending in the *b*-axis direction.

## Related literature

For mixed-valence copper cyanide complexes crystallizing as one- two- and three-dimensional self-assembled polymeric networks involving cyanide groups bridging copper atoms, see: Williams *et al.* (1972 ▶); Colacio *et al.* (2002 ▶); Kim *et al.* (2005 ▶). For discrete mol­ecules containing terminal cyanide groups which are not involved in any covalent polymeric linkages, see: Yuge *et al.* (1998 ▶); Pickardt *et al.* (1999 ▶); Pretsch *et al.* (2005 ▶). For the structure of a related one-dimensional polymer, see: Corfield & Yang (2012 ▶). For cyanide analysis, see: Cooper & Plane (1966 ▶).
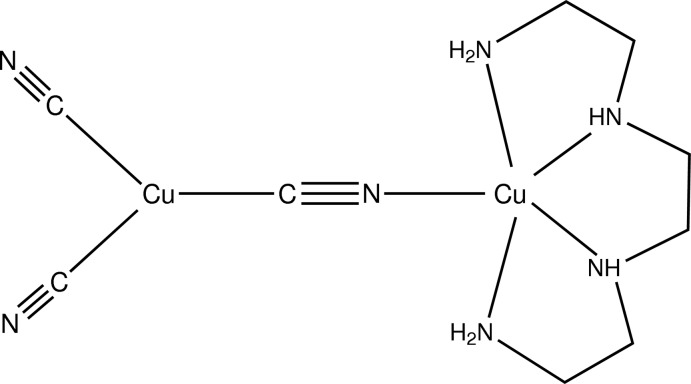


## Experimental

### 

#### Crystal data

[Cu_2_(CN)_3_(C_6_H_18_N_4_)]
*M* *_r_* = 351.38
Triclinic, 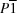

*a* = 7.363 (3) Å
*b* = 8.741 (6) Å
*c* = 11.492 (6) Å
α = 77.84 (3)°
β = 73.78 (3)°
γ = 83.18 (3)°
*V* = 692.8 (7) Å^3^
*Z* = 2
Cu *K*α radiation
μ = 3.75 mm^−1^
*T* = 295 K
0.7 × 0.2 × 0.1 mm

#### Data collection

GE 1/4 circle manual diffractometer
Absorption correction: integration (Busing & Levy, 1957 ▶) *T*_min_ = 0.515, *T*_max_ = 0.746
2734 measured reflections
2060 independent reflections
1975 reflections with *I* > 2σ(*I*)
*R*_int_ = 0.042
θ_max_ = 60.0°
3 standard reflections every 22 reflections intensity decay: 0.2 (2)%

#### Refinement

*R*[*F* ^2^ > 2σ(*F* ^2^)] = 0.039
*wR*(*F* ^2^) = 0.120
*S* = 1.16
2060 reflections
163 parameters
H-atom parameters constrained
Δρ_max_ = 0.51 e Å^−3^
Δρ_min_ = −0.56 e Å^−3^

### 

Data collection: locally modified program (Corfield, 1984 ▶); cell refinements and data reduction followed procedures described by Corfield *et al.* (1973 ▶); data were averaged with *SORTAV* (Blessing, 1989 ▶); program(s) used to solve structure: locally modified program (Corfield, 1984 ▶); program(s) used to refine structure: *SHELXL97* (Sheldrick, 2008 ▶) and *XABS2* (Parkin *et al.*, 1995 ▶); molecular graphics: *ORTEPIII* (Burnett & Johnson, 1996 ▶); software used to prepare material for publication: *SHELXL97*.

## Supplementary Material

Crystal structure: contains datablock(s) I, global. DOI: 10.1107/S1600536812047745/lh5552sup1.cif

Structure factors: contains datablock(s) I. DOI: 10.1107/S1600536812047745/lh5552Isup2.hkl

Additional supplementary materials:  crystallographic information; 3D view; checkCIF report

## Figures and Tables

**Table 1:** Selected bond lengths (Å)

Cu1—C1	1.947 (4)
Cu1—C2	1.925 (4)
Cu1—C3	1.948 (5)
Cu2—N1	2.127 (4)
Cu2—N4	2.045 (4)
Cu2—N7	2.034 (4)
Cu2—N10	2.047 (4)
Cu2—N13	2.028 (4)

**Table 2:** Hydrogen-bond geometry (Å, °)

*D*—H⋯*A*	*D*—H	H⋯*A*	*D*⋯*A*	*D*—H⋯*A*
N7—H7⋯N2^i^	0.91	2.14	2.984 (6)	154
N10—H10⋯N3^ii^	0.91	2.28	3.178 (6)	171

Symmetry codes: (i) 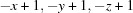; (ii) 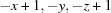.

**Fig. 1.**
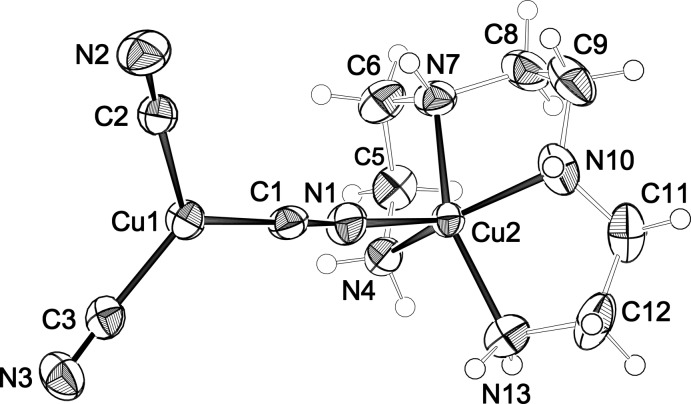
The molecular structure of the [Cutrien(CN)Cu(CN)2] molecule, with ellipsoids at the 50% level.

**Fig. 2.**
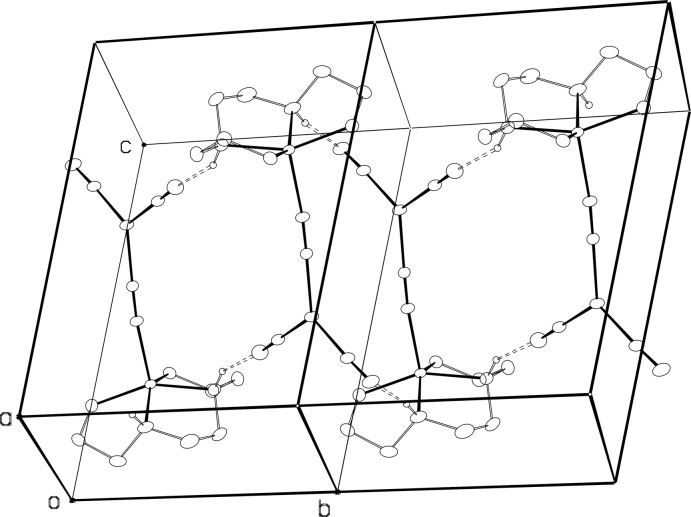
Packing of [Cutrien(CN)Cu(CN)2], viewed along the a axis, showing the main hydrogen bonds as dashed lines.

## supplementary crystallographic information

### Comment

The structure determination of the title compound was undertaken as part of a series of synthetic and structural studies of mixed-valence copper cyanide complexes containing amine bases. The coordinated amines stabilize the divalent copper atoms against reduction by the cyanide groups. In this study, the synthesis involved the linear tetradentate base triethylenetetramine (trien), under conditions expected to produce a polymeric structure, as in Williams *et al.* (1972); Colacio *et al.* (2002); Kim *et al.* (2005). Instead the crystal structure is made up of discrete molecules with terminal cyanide groups that are not involved in covalent polymeric linkages, and is similar to structures reported by: Yuge *et al.* (1998); Pickardt *et al.* (1999); Pretsch *et al.* (2005).

The molecules contain a divalent and a monovalent copper atom bridged by a cyanide group. The divalent copper atom, Cu2, shows square-pyramidal coordination, with the four N atoms of the tetradentate ligand occupying the basal positions, and the N atom of the bridging cyanide group in the apical position. Cu2 lies 0.490 (2) Å out of the best plane through the four nitrogen atoms of the ligand, in the direction of the apical N atom. The three Cu—N—C—C—N—Cu chelate rings have torsional angles of 49.9 (5) °, 51.7 (6) ° and 53.1 (6) °. The monovalent copper atom, Cu1, shows trigonal planar coordination to the carbon atoms of the bridging and two terminal cyanide groups, with bond angles close to 120 ° and Cu1 almost coplanar with the three cyanide carbon atoms, lying 0.039 (3) Å above their plane.

The bridging C—N bond length of 1.131 (6) Å is not significantly different from the terminal C—N bond lengths. This C—N group is linearly bonded to the two copper atoms, with the angle Cu1—C—N = 177.9 (4)° and C—N—Cu2 = 174.0 (4)°. The linear geometry differs from that found in the one-dimensional polymer [Cu(dien)CN]^+^, (Corfield & Yang, 2012) where both copper atoms are divalent, and the C—N—Cu angle is non-linear at 146.5 (2)°. The Jahn-Teller lengthening of the axial Cu—N distance is greater in the polymer, with Cu—N = 2.340 (3) Å *versus* 2.127 (4) Å in the present structure.

Two prominent hydrogen bonds are seen linking N—H bonds from the trien ligand and nitrogen atoms of neighboring terminal cyanide groups. The N7—H7.. . N2(1 - *x*,1 - *y*,1 - *z*) hydrogen bond joins the two molecules in the unit cell into a centrosymmetric dimer, while the N10—H10···N3(1 - *x*,-*y*,1 - *z*) hydrogen bonds link these dimers into a ribbon extending along the direction of the *b* axis, as shown in figure 2. Hydrogen bonds between terminal cyanide groups and N—H bonds are also seen in the similar compound studied by Pretsch *et al.* (2005).

Intermolecular contacts appear normal, with the shortest H—H intermolecular distance found at 2.53 Å. There are short contacts, however, between the C atoms of the terminal cyanide groups and H atoms of neighboring molecules, with C3—H13B(-*x*, -*y*, 1 - *z*) at 2.42 Å, and C2—H4B(1 + *x*, *y*, *z*) at 2.55 Å.

### Experimental

The compound was prepared by addition of 20 ml of a solution containing 0.020 mol of CuSO_4_^.^5H_2_O and 0.020 mol of trien to 20 ml of a solution containing 0.040 mol of NaCN. A yield of approximately 1.0 g of blue crystals in the form of diamond plates elongated along b was obtained. Total copper was analyzed iodometrically: found 35.7%; calculated 36.2%. Cyanide was analyzed by AgNO_3_ titration of the HCN gas evolved on addition of 15*M* HNO_3_ to a sample, as in Cooper & Plane (1966): found 20.3%; calculated 22.2%. This corresponds to a recovery rate of 91%, which was typical for our test analyses by this method. CN stretching frequencies were found at 2089 (s, doublet) and 2110 (*m*) cm^-1^, with a Perkin-Elmer 710B spectrophotometer.

### Refinement

All 18 hydrogen atoms of the diethylenetriamine ligand were found unambiguously from a difference Fourier map, and were initially refined freely. In the final refinements, hydrogen atoms were constrained to idealized positions by *SHELXL* (Sheldrick, 2008). No improvement was found by refining the NH atoms independently.

Refinements with anisotropic temperature factors for Cu, N and C atoms and constrained hydrogen atom parameters converged smoothly, but a difference Fourier synthesis at this stage showed a pattern of peaks and holes of 1.0–1.2 e/A^3^ associated with the copper atoms. Further, examination of the observed and calculated structure factors showed a clear anisotropic variation in scale factor. This anisotropy was modeled by using the program *XABS2*, (Parkin *et al.*, 1995) to modify the observed structure factors. The structure presented here is based upon subsequent refinements in *SHELXL* (Sheldrick, 2008) that lowered *R*_1_ from 0.0544 to 0.0402 for all reflections and *R*_2_ from 0.1552 to 0.1201. Changes in atomic parameters were small: the maximum shift was 1.6σ, with only 22 of the 162 atomic parameters changing by more than 1σ. The anisotropy in the scale factor and the noise in the final difference Fourier map were both lowered.

There is no evidence of disorder between the C and N atoms of the cyanide groups. Assignments of C and N atoms in the cyanide groups were verified after the last stage of the refinement, when the C and N atoms for each group were in turn inverted. In each case, *R* values increased, and the U values became unlikely.

### Crystal data

**Table:**

[Cu_2_(CN)_3_(C_6_H_18_N_4_)]	*Z* = 2
*M**_r_* = 351.38	*F*(000) = 358
Triclinic, *P*1	*D*_x_ = 1.684 Mg m^−^^3^*D*_m_ = 1.684 (2) Mg m^−^^3^*D*_m_ measured by flotation in 1,2-dibromoethane/carbon tetrachloride mixtures
Hall symbol: -P 1	Cu *K*α radiation, λ = 1.5418 Å
*a* = 7.363 (3) Å	Cell parameters from 16 reflections
*b* = 8.741 (6) Å	θ = 23.5–41°
*c* = 11.492 (6) Å	µ = 3.75 mm^−^^1^
α = 77.84 (3)°	*T* = 295 K
β = 73.78 (3)°	Diamond plate, blue
γ = 83.18 (3)°	0.7 × 0.2 × 0.1 mm
*V* = 692.8 (7) Å^3^	

### Data collection

**Table:**

GE 1/4 circle manual diffractometer	1975 reflections with *I* > 2σ(*I*)
Radiation source: sealed X-ray tube	*R*_int_ = 0.042
None monochromator	θ_max_ = 60.0°, θ_min_ = 4.1°
θ/2θ scans	*h* = −8→8
Absorption correction: integration (Busing & Levy, 1957)	*k* = 0→9
*T*_min_ = 0.515, *T*_max_ = 0.746	*l* = −12→12
2734 measured reflections	3 standard reflections every 22 reflections
2060 independent reflections	intensity decay: 0.2(2)

### Refinement

**Table:**

Refinement on *F*^2^	Primary atom site location: heavy-atom method
Least-squares matrix: full	Secondary atom site location: difference Fourier map
*R*[*F*^2^ > 2σ(*F*^2^)] = 0.039	Hydrogen site location: difference Fourier map
*wR*(*F*^2^) = 0.120	H-atom parameters constrained
*S* = 1.16	*w* = 1/[σ^2^(*F*_o_^2^) + (0.035*P*)^2^ + 1.1*P*] where *P* = (*F*_o_^2^ + 2*F*_c_^2^)/3
2060 reflections	(Δ/σ)_max_ < 0.001
163 parameters	Δρ_max_ = 0.51 e Å^−^^3^
0 restraints	Δρ_min_ = −0.56 e Å^−^^3^

### Special details

**Table:**

Experimental. crystal A: 2θ 0–35°; 32–50°; 47–79°. small crystal, showing visible decomposition after data collection, with 13% loss of intensities; no absorption correction. crystal B: 2θ 0–40°; 65–90°; 86–120°. larger crystal, mounted in capillary tube; no fall-off of intensities of standard reflections, but intensities fluctuated with an e.s.d. of 3%.
Geometry. All e.s.d.'s (except the e.s.d. in the dihedral angle between two l.s. planes) are estimated using the full covariance matrix. The cell e.s.d.'s are taken into account individually in the estimation of e.s.d.'s in distances, angles and torsion angles; correlations between e.s.d.'s in cell parameters are only used when they are defined by crystal symmetry. An approximate (isotropic) treatment of cell e.s.d.'s is used for estimating e.s.d.'s involving l.s. planes.
Refinement. Refinement of *F*^2^ against ALL reflections. The weighted *R*-factor *wR* and goodness of fit *S* are based on *F*^2^, conventional *R*-factors *R* are based on *F*, with *F* set to zero for negative *F*^2^. The threshold expression of *F*^2^ > σ(*F*^2^) is used only for calculating *R*-factors(gt) *etc*. and is not relevant to the choice of reflections for refinement. *R*-factors based on *F*^2^ are statistically about twice as large as those based on *F*, and *R*- factors based on ALL data will be even larger.

### Fractional atomic coordinates and isotropic or equivalent isotropic displacement parameters (Å^2^)

**Table:**

	*x*	*y*	*z*	*U*_iso_*/*U*_eq_	
Cu1	0.40015 (8)	0.09959 (7)	0.66481 (5)	0.0371 (2)	
Cu2	0.09442 (7)	0.23562 (6)	0.29634 (5)	0.0288 (2)	
N1	0.2352 (5)	0.1692 (4)	0.4395 (3)	0.0419 (9)	
C1	0.2989 (6)	0.1424 (5)	0.5210 (4)	0.0361 (9)	
N2	0.6787 (7)	0.3236 (5)	0.6847 (4)	0.0591 (11)	
C2	0.5694 (6)	0.2419 (5)	0.6797 (4)	0.0367 (9)	
N3	0.2855 (6)	−0.1824 (5)	0.8742 (4)	0.0518 (10)	
C3	0.3224 (6)	−0.0759 (5)	0.7997 (4)	0.0375 (9)	
N4	−0.1840 (5)	0.2330 (4)	0.3945 (3)	0.0388 (8)	
H4A	−0.2410	0.1593	0.3758	0.047*	
H4B	−0.1926	0.2104	0.4759	0.047*	
C5	−0.2766 (6)	0.3882 (5)	0.3625 (4)	0.0442 (10)	
H5A	−0.3962	0.4010	0.4231	0.066*	
H5B	−0.3011	0.4006	0.2823	0.066*	
C6	−0.1434 (7)	0.5083 (6)	0.3605 (5)	0.0487 (11)	
H6A	−0.1897	0.6119	0.3264	0.073*	
H6B	−0.1388	0.5085	0.4440	0.073*	
N7	0.0470 (5)	0.4715 (4)	0.2857 (3)	0.0390 (8)	
H7	0.1343	0.5080	0.3138	0.047*	
C8	0.0793 (7)	0.5320 (6)	0.1524 (5)	0.0511 (12)	
H8A	0.0739	0.6457	0.1359	0.077*	
H8B	−0.0171	0.4986	0.1220	0.077*	
C9	0.2683 (7)	0.4689 (7)	0.0909 (5)	0.0579 (13)	
H9A	0.3649	0.5165	0.1115	0.087*	
H9B	0.2873	0.4950	0.0022	0.087*	
N10	0.2870 (5)	0.2968 (5)	0.1299 (3)	0.0438 (9)	
H10	0.4064	0.2669	0.1376	0.053*	
C11	0.2395 (8)	0.2055 (7)	0.0501 (4)	0.0581 (13)	
H11A	0.3345	0.2151	−0.0285	0.087*	
H11B	0.1175	0.2438	0.0349	0.087*	
C12	0.2329 (9)	0.0378 (7)	0.1147 (5)	0.0619 (15)	
H12A	0.1878	−0.0240	0.0688	0.093*	
H12B	0.3590	−0.0042	0.1199	0.093*	
N13	0.1033 (5)	0.0289 (4)	0.2408 (3)	0.0412 (8)	
H13A	0.1450	−0.0496	0.2934	0.049*	
H13B	−0.0137	0.0092	0.2402	0.049*	

### Atomic displacement parameters (Å^2^)

**Table:**

	*U*^11^	*U*^22^	*U*^33^	*U*^12^	*U*^13^	*U*^23^
Cu1	0.0395 (4)	0.0409 (4)	0.0366 (4)	−0.0088 (3)	−0.0201 (3)	−0.0029 (3)
Cu2	0.0309 (3)	0.0298 (3)	0.0279 (3)	−0.0016 (2)	−0.0120 (2)	−0.0049 (2)
N1	0.052 (2)	0.040 (2)	0.041 (2)	−0.0007 (16)	−0.0275 (18)	−0.0062 (16)
C1	0.047 (2)	0.028 (2)	0.039 (2)	−0.0068 (17)	−0.020 (2)	−0.0054 (17)
N2	0.064 (3)	0.057 (3)	0.068 (3)	−0.017 (2)	−0.030 (2)	−0.011 (2)
C2	0.040 (2)	0.038 (2)	0.036 (2)	−0.0038 (19)	−0.0168 (18)	−0.0039 (17)
N3	0.054 (2)	0.057 (3)	0.044 (2)	−0.009 (2)	−0.0183 (18)	0.004 (2)
C3	0.034 (2)	0.046 (2)	0.038 (2)	−0.0031 (18)	−0.0189 (18)	−0.007 (2)
N4	0.0410 (19)	0.0398 (19)	0.0367 (18)	−0.0065 (15)	−0.0072 (15)	−0.0113 (15)
C5	0.033 (2)	0.050 (3)	0.050 (3)	0.0004 (19)	−0.0089 (19)	−0.015 (2)
C6	0.050 (3)	0.043 (3)	0.058 (3)	0.007 (2)	−0.017 (2)	−0.020 (2)
N7	0.044 (2)	0.0341 (18)	0.045 (2)	−0.0091 (15)	−0.0206 (16)	−0.0046 (15)
C8	0.062 (3)	0.039 (2)	0.053 (3)	−0.007 (2)	−0.026 (2)	0.009 (2)
C9	0.050 (3)	0.068 (3)	0.047 (3)	−0.023 (2)	−0.009 (2)	0.012 (2)
N10	0.0299 (17)	0.060 (2)	0.0361 (19)	0.0003 (16)	−0.0094 (14)	0.0017 (17)
C11	0.063 (3)	0.077 (4)	0.033 (2)	0.006 (3)	−0.013 (2)	−0.013 (2)
C12	0.082 (4)	0.063 (3)	0.049 (3)	0.025 (3)	−0.028 (3)	−0.032 (3)
N13	0.0366 (18)	0.041 (2)	0.052 (2)	0.0040 (15)	−0.0192 (16)	−0.0150 (17)

### Geometric parameters (Å, º)

**Table:**

Cu1—C1	1.947 (4)	C6—H6B	0.9700
Cu1—C2	1.925 (4)	N7—C8	1.472 (6)
Cu1—C3	1.948 (5)	N7—H7	0.9100
Cu2—N1	2.127 (4)	C8—C9	1.474 (8)
Cu2—N4	2.045 (4)	C8—H8A	0.9700
Cu2—N7	2.034 (4)	C8—H8B	0.9700
Cu2—N10	2.047 (4)	C9—N10	1.478 (7)
Cu2—N13	2.028 (4)	C9—H9A	0.9700
C1—N1	1.131 (6)	C9—H9B	0.9700
C2—N2	1.158 (6)	N10—C11	1.468 (7)
C3—N3	1.126 (6)	N10—H10	0.9100
N4—C5	1.467 (6)	C11—C12	1.497 (8)
N4—H4A	0.9000	C11—H11A	0.9700
N4—H4B	0.9000	C11—H11B	0.9700
C5—C6	1.511 (7)	C12—N13	1.488 (6)
C5—H5A	0.9700	C12—H12A	0.9700
C5—H5B	0.9700	C12—H12B	0.9700
C6—N7	1.464 (6)	N13—H13A	0.9000
C6—H6A	0.9700	N13—H13B	0.9000
			
C1—Cu1—C2	119.26 (17)	C8—N7—H7	109.4
C2—Cu1—C3	118.75 (17)	Cu2—N7—H7	109.4
C3—Cu1—C1	121.87 (17)	N7—C8—C9	107.4 (4)
N1—Cu2—N4	101.66 (15)	N7—C8—H8A	110.2
N1—Cu2—N7	102.70 (14)	C9—C8—H8A	110.2
N1—Cu2—N10	110.18 (15)	N7—C8—H8B	110.2
N1—Cu2—N13	101.54 (15)	C9—C8—H8B	110.2
N4—Cu2—N7	83.37 (15)	H8A—C8—H8B	108.5
N7—Cu2—N10	83.65 (16)	C8—C9—N10	110.6 (4)
N10—Cu2—N13	84.25 (16)	C8—C9—H9A	109.5
N4—Cu2—N13	95.70 (15)	N10—C9—H9A	109.5
N7—Cu2—N13	155.42 (15)	C8—C9—H9B	109.5
N4—Cu2—N10	147.54 (15)	N10—C9—H9B	109.5
C1—N1—Cu2	174.0 (4)	H9A—C9—H9B	108.1
Cu1—C1—N1	177.9 (4)	C11—N10—C9	115.3 (4)
Cu1—C2—N2	176.5 (4)	C11—N10—Cu2	103.8 (3)
Cu1—C3—N3	176.1 (4)	C9—N10—Cu2	108.3 (3)
C5—N4—Cu2	108.3 (3)	C11—N10—H10	109.7
C5—N4—H4A	110.0	C9—N10—H10	109.7
Cu2—N4—H4A	110.0	Cu2—N10—H10	109.7
C5—N4—H4B	110.0	N10—C11—C12	107.7 (4)
Cu2—N4—H4B	110.0	N10—C11—H11A	110.2
H4A—N4—H4B	108.4	C12—C11—H11A	110.2
N4—C5—C6	107.1 (4)	N10—C11—H11B	110.2
N4—C5—H5A	110.3	C12—C11—H11B	110.2
C6—C5—H5A	110.3	H11A—C11—H11B	108.5
N4—C5—H5B	110.3	N13—C12—C11	109.0 (4)
C6—C5—H5B	110.3	N13—C12—H12A	109.9
H5A—C5—H5B	108.5	C11—C12—H12A	109.9
N7—C6—C5	110.1 (4)	N13—C12—H12B	109.9
N7—C6—H6A	109.6	C11—C12—H12B	109.9
C5—C6—H6A	109.6	H12A—C12—H12B	108.3
N7—C6—H6B	109.6	C12—N13—Cu2	109.1 (3)
C5—C6—H6B	109.6	C12—N13—H13A	109.9
H6A—C6—H6B	108.1	Cu2—N13—H13A	109.9
C6—N7—C8	115.0 (4)	C12—N13—H13B	109.9
C6—N7—Cu2	110.0 (3)	Cu2—N13—H13B	109.9
C8—N7—Cu2	103.4 (3)	H13A—N13—H13B	108.3
C6—N7—H7	109.4		
			
N4—C5—C6—N7	49.9 (5)	N10—C11—C12—N13	53.1 (6)
N7—C8—C9—N10	51.7 (6)		

### Hydrogen-bond geometry (Å, º)

**Table:**

*D*—H···*A*	*D*—H	H···*A*	*D*···*A*	*D*—H···*A*
N7—H7···N2^i^	0.91	2.14	2.984 (6)	154
N10—H10···N3^ii^	0.91	2.28	3.178 (6)	171

Symmetry codes: (i) −*x*+1, −*y*+1, −*z*+1; (ii) −*x*+1, −*y*, −*z*+1.
